# Development of the non-occupational post-exposure prophylaxis (NPEP) knowledge scale among Chinese men who have sex with men

**DOI:** 10.1186/s12889-023-16206-5

**Published:** 2023-07-11

**Authors:** Haochu Li, Ran Wei, Traci L. Weinstein, Eunsook Kim, Angela J. Jacques-Tiura, Xiaofu Ning, Wei Ma

**Affiliations:** 1 Department of Epidemiology, School of Public Health, Shandong University, 44 West Wenhua Road, Jinan, 250012 Shandong Province China; 2 Department of Psychology, Rhode Island College, Providence, RI USA; 3 Department of Educational and Psychological Studies, University of South Florida, Tampa, FL USA; 4 Department of Family Medicine and Public Health Sciences, Wayne State University, Detroit, MI USA; 5 School of Pharmaceutical Sciences, Peking University, Beijing, China

**Keywords:** Nonoccupational post-exposure Prophylaxis (NPEP), Psychometrics, Structural equation modeling, Men who have sex with men, China

## Abstract

**Background:**

Nonoccupational post-exposure prophylaxis (NPEP) is a short course of medication taken to reduce the likelihood of acquiring human immunodeficiency virus (HIV) infection upon exposure. A review of the literature demonstrates an urgent need for an empirically validated instrument that measures detailed knowledge of NPEP among the key population of men who have sex with men (MSM).

**Methods:**

Semi-structured interviews, focus groups, and a cross-sectional survey with a sample of 419 MSM was conducted in 2018 in China to develop and psychometrically evaluate the new instrument, the NPEP Knowledge Scale. Exploratory and confirmatory factor analyses, differential item functioning analyses, and structural equation modeling were conducted using Mplus 7.4.

**Results:**

The NPEP Knowledge Scale demonstrated excellent reliability and validity. Cronbach’s alpha was 0.903. The range of item R^2^ were 0.527–0.969, *p*’s < 0.001. Model estimated inter-item correlations ranged between 0.534 and 0.968. In addition, HIV knowledge, NPEP use, and NPEP knowledge were all significantly correlated.

**Conclusions:**

The NPEP Knowledge Scale is suitable for research, program evaluation, and clinical and community services that require using NPEP to minimize the ever-present risk of new HIV infections.

**Supplementary Information:**

The online version contains supplementary material available at 10.1186/s12889-023-16206-5.

The human immunodeficiency virus (HIV) epidemic among men who have sex with men (MSM) is still a significant national public health challenge in China. A recent large-scale systematic analysis reported an estimated national prevalence of HIV from 2001 to 2018 of 5.7% (95% confidence interval [CI]: 5.4–6.1%) among Chinese MSM, and those MSM aged 50 years and older had the highest HIV prevalence of 19.3% (95% CI: 13.1–27.4%), compared with other age groups [1].

Post-exposure prophylaxis (PEP), a four-week course of antiretroviral medications, is currently the best HIV intervention method when someone is potentially exposed [2]. The research on PEP generally focuses on occupational use for HIV-exposed health care workers. In contrast, the research on nonoccupational post-exposure prophylaxis (NPEP) focuses on people with high likelihood of exposure to HIV outside of occupational realms (e.g., through sexual activity or needle sharing). Although most recent reviews detail how NPEP can be useful in curtailing the ever-present risk of new HIV infections, NPEP education and awareness has remained low since its inception [3, 4]. One study reported only about 41% of MSM surveyed from Boston, Pittsburgh, and San Juan indicated having heard of PEP, and less than 1% had used it [5]. In China, PEP has not been officially approved for non-occupational use, and not surprisingly then, NPEP is underutilized [6]. More awareness of NPEP may help many more people to gain access and prevent HIV infection [7]. This investigation of NPEP in China is therefore fully justified.

Although the Knowledge, Attitude and Practices (KAP) survey has been widely used in health behavior research on HIV, assessments of NPEP knowledge and use are yet limited. Thus, it is necessary for researchers to develop a specific and effective tool to examine the knowledge of NPEP. Current tools assessing detailed NPEP knowledge are limited and typically consist of minimal, individual-prompted items [8–11]. Outside of occupational use, among patients and community members specifically, past researchers have used tools that assessed: (1) access to NPEP information or awareness of NPEP availability [8]; (2) awareness of, or willingness to use, NPEP [9], and (3) intentions to use NPEP [10]. A recent systematic review examined only rates of provision of NPEP by health care workers, and rates of acceptance and completion by patients, but not NPEP knowledge [12]. The current tools are lacking assessment of in-depth PEP knowledge, particularly outside of occupational use (i.e., regarding NPEP).

We are aware of only two studies reporting NPEP awareness and services in China. The first paper discussed NPEP services in an HIV/AIDS designated hospital in Beijing [13], and the second study found that only 22% of Chinese MSM had heard about NPEP [14]. However, neither study assessed detailed NPEP knowledge or factors related to NPEP knowledge (e.g., age, HIV knowledge, NPEP use).

Taken together, the literature indicates that development of a detailed NPEP knowledge instrument appears to be much needed and long overdue. The aim of this study was to develop a valid and reliable measurement tool to assess detailed NPEP knowledge. We expected that items in our newly developed NPEP Knowledge Scale would demonstrate good internal reliability, with consistent responding patterns among participants. We also expected to find evidence of convergent validity (i.e., that NPEP knowledge would be positively associated with HIV knowledge), criterion validity (i.e., that NPEP knowledge would be positively associated with NPEP use), and significant relationships between the NPEP Knowledge Scale scores and sociodemographic and behavioral factors.

## Method

### Participants and procedures

We conducted a two-phased, mixed-methods, cross-sectional study to develop and validate the NPEP Knowledge Scale among Chinese MSM in 2018. We selected two big cities with large, accessible MSM populations: Shijiazhuang in northern China and Xiamen in southern China. Local community-based organizations (CBOs) and the Chinese Center for Disease Control (China CDC) helped with the data collection. Inclusion criteria for the survey participants were: being male and aged 18 years and over; having had sex with another male in the past 12 months; self-reporting being HIV negative or unaware of HIV infection status; and being willing to provide written informed consent and voluntarily participate in the survey, interview, or focus group. Exclusion criteria were: self-reporting HIV positive status; not being able to complete the survey, interview, or focus group due to health problems (e.g., mental illness, consciousness disorder); and not being able to provide written informed consent. We completed a written informed consent process introducing participants to the study’s purpose and contents, and emphasizing confidentiality (and anonymity for survey participants) before conducting qualitative interviews, focus groups, or the quantitative survey.

#### Phase 1: formative qualitative work to develop NPEP knowledge scale items

We first drafted a qualitative interview protocol based on the research literature, then experts revised it, and we finalized it after research group discussions. Using the developed protocol, we conducted face-to-face, semi-structured, in-depth interviews with 21 MSM. In the sample, some MSM who had used NPEP were purposively recruited to better understand their real-world experiences of using NPEP. Primary interview questions assessed participants’ knowledge of, needs for, perceived risks of using, and willingness to use NPEP; attitudes toward and suggestions for NPEP services; and possible impacts of NPEP on changing risk behaviors.

Additionally, we recruited 11 health care workers with NPEP provision experience to participate in focus group discussions. Primary discussion prompts concerned difficulties encountered in implementing/planning NPEP services, MSM populations’ concerns about NPEP, suggestions for NPEP services among MSM, and possible impacts of NPEP on risk behavior change. For both interviews and focus groups, we were specifically interested in looking at themes related to NPEP knowledge in the current study.

We conducted the in-depth interviews at CBOs, and we conducted the focus group discussions in local AIDS-designated hospitals. After participants provided written informed consent, the interview or focus group was started and recorded. Interviews lasted 30–60 min; focus group discussions lasted 80–105 min. Participants were provided subsidies (CNY ¥ 100, about $15 USD) to compensate them for their time and transportation.

The interview and focus group recordings were transcribed verbatim and encoded using ATLAS.ti 5.0 software. Thematic content analysis was conducted using the interview and focus group guides as a coding framework. According to the results of the qualitative study, the quantitative survey questionnaire was developed. The qualitative findings guided the NPEP Knowledge Scale item content and phrasing.

#### Phase 2: survey using the NPEP knowledge scale

We next conducted a quantitative survey that included the newly developed quantitative NPEP Knowledge Scale. Participants received a questionnaire link from Wenjuanxing (a professional electronic survey platform) and personal codes. The use of a personal survey code was to ensure better authenticity of survey results. Participants completed the survey independently on their phone or computer. The research team reviewed submitted questionnaires and then provided participants CNY ¥ 30 (about $4.50 USD) through Wechat Red Pack (a free messaging and calling app) as compensation. We received a total of 423 questionnaires and retained 419 for data analysis after four questionnaires were deleted due to logic errors. Sample socio-demographic information and HIV-related behaviors and service utilization are presented in Table 1.

**Table 1:** Participants’ socio-demographic and behavioral characteristics (n = 419 Chinese MSM)

	Number	Percent
Age (*M* = 28.04, *SD* = 9.71)		
18–34	330	78.8
35–49	69	16.4
50–64	19	4.6
65+	1	0.2
Ethnicity		
Han	407	97.1
Other ethnicity	12	2.9
Duration of local residence		
< 3 months	22	5.3
3–6 months	22	5.3
Above 6–12 months	20	4.8
Above 12–24 months	60	14.3
Above 24 months	295	70.4
Education		
Lower than primary school	4	1.0
Primary school	5	1.2
Secondary school	47	11.2
High/Polytechnic school	87	20.8
College or above	276	65.9
Monthly income (CNY ¥)*		
<1500	89	21.2
1500–3000	72	17.2
3001–5000	123	29.4
5001–8000	75	17.9
>8000	49	11.7
missing	11	2.6
Marital status with female		
never married	322	76.8
married	68	16.2
cohabit	13	3.1
divorced/separated/widowed	16	3.8
Sexual orientation		
homosexual	260	62.1
bisexual	106	25.3
heterosexual	16	3.8
uncertain	37	8.8
Number of incidences of having anal sex with males in the last week		
0	245	58.5
1–4	162	38.7
5–8	10	2.4
>8	2	0.5
Number of male sexual partners in the last 6 months		
0	127	30.3
1	121	28.9
2–6	146	34.8
≥7	25	6.0
Condom use in the most recent anal sex with other males in the last 6 months		
Yes	241	57.5
No	51	12.2
No anal sex	127	30.3
Condom rupture, slippage or intentional pull-out when having sex with other males in the last 6 months		
Yes	23	5.5
No	253	60.4
unknown	16	3.8
No anal sex	127	30.3
Use of illicit drugs		
Yes	33	7.9
No	386	92.1
Frequency of drinking alcohol in the last 3 months		
0	147	35.1
1–2 times	127	30.3
1–3 times per month	91	21.7
1–4 times per week	39	9.3
5–7 times or above per week	15	3.6
Diagnosis of STI in the past 12 months		
Yes	22	5.3
No	397	94.7
Frequency of HIV testing		
Never	77	18.4
Tested in 12 months ago	50	11.9
Tested once in the last 12 months	136	32.5
Tested for 2 or more times in the last 12 months	156	37.2
Use of NPEP		
Yes	15	3.6
No	404	96.4

* $1 USD is approximately equivalent to CNY ¥ 6.667

### Survey measures

#### NPEP knowledge scale

Guided by the Phase 1 formative work, the NPEP Knowledge Scale included 11 items assessing key NPEP knowledge points. The original response options included *correct*, *incorrect*, and *unknown*, which were collapsed into binary items data analysis (with *unknown* treated as *incorrect*). The scale score was computed as the sum of correct answers, thus higher scores indicated better NPEP knowledge. The scale was administered in Chinese (see Table 2 for the English translation of items).

**Table 2:** Exploratory factor analysis structure coefficients and extracted communalities for items assessed for the NPEP knowledge scale among Chinese MSM, 2018 (n = 219)

Scale items	Communalities (h^2^)	Geomin Rotated loadings	Percentages answering correctly
A. NPEP can be taken within one week after the occurrence of behaviors that may lead to the infection of HIV (e.g., unprotected sex, sharing needles) ^a^	0.841	0.830	47.5%
B. A person does not need to take NPEP after having unprotected sex with people with unknown HIV status ^a^	0.958	0.966	64.4%
C. A person needs to take NPEP after sharing a needle with people living with HIV or with unknown HIV status	0.968	0.975	66.3%
D. A person needs to take NPEP when one’s mucosa or damaged skin is exposed to the blood or other body fluids of people living with HIV	0.956	1.004	68.3%
E. A person needs to make sure they are not infected with HIV before taking NPEP	0.590	0.744	41.5%
F. A person needs to take NPEP even though condoms were used correctly throughout the process of sexual activities, regardless of the HIV status of sexual partners ^a^	0.760	0.895	52.3%
G. A person does not need to take NPEP if the source of exposure (e.g., sexual partners, peers of injection drug users, etc.) is not found to be infected with HIV	0.534	0.823	44.9%
H. The course of NPEP treatment is 28 days. Intermittent medication can be used as long as a cumulative use of NPEP lasts for 28 days ^a^	0.801	0.913	53.0%
I. Relevant tests are needed before, during and after taking NPEP	0.974	0.961	64.2%
J. NPEP can be taken within 72 h after the occurrence of behaviors that may lead to the infection of HIV, and the sooner the better	0.986	1.013	67.1%
K. When taking NPEP, there may be different degrees of drug reactions (e.g., dizziness, nausea, diarrhea, fever, rash, liver and kidney damage) due to different personal physical conditions. NPEP users can stop taking NPEP independently according to their own drug reactions ^a^	0.679	0.769	34.1%

*Note*. The EFA model has acceptable fit indices (RMSEA = 0.061, 90% CI 0.038–0.082, CFI = 0.998, TLI = 0.998). All Geomin rotated loadings in Table 2 are significant at 5% level. The final version of the scale omits items C and J. ^a^ Reversed item

#### HIV knowledge

We used a popular 8-question scale developed by the China CDC to assess HIV knowledge [15]. Response options were *yes*, *no*, and *unknown*, which were then transformed into *correct* or *incorrect* (including *unknown*) binary items for data analysis. The scale score was computed as the sum of correct answers, thus higher scores indicated better HIV knowledge. Latent variable modeling showed that the HIV Knowledge Scale’s reliability was 0.824 (95% CI 0.796, 0.853). The overall mean score was 6.77 (standard deviation [SD] = 1.56).

#### HIV-related behaviors and utilization of services

We assessed HIV-related behaviors and service utilization with nine items, shown in Table 1. These items included number of incidences of having anal sex with males in the last week; number of male sexual partners in the last 6 months; condom use in the most recent anal sex with other males in the last 6 months; condom rupture, slippage, or intentional pull-out when having sex with other males in the last 6 months; illicit drug use; frequency of drinking alcohol in the last 3 months; sexually transmitted infection (STI) diagnosis in the past 12 months; frequency of HIV testing; and NPEP use.

#### Socio-demographic information

Also shown in Table 1, we included seven items assessing participants’ socio-demographic characteristics. These items included age, ethnicity, duration of local residence, education, monthly income, marital status with females, and sexual orientation.

### Quantitative statistical analysis

We first conducted descriptive analyses. We then randomly split the sample into two subsamples for subsequent exploratory factor analysis (EFA) and confirmatory factor analysis (CFA) in SPSS 19. We then used independent-samples t-tests and chi-square tests to ensure that there were no significant differences between the two subsamples in terms of sociodemographic or behavioral characteristics.

We next used Mplus 7.4 to conduct EFA, CFA, and structural equation modeling (SEM). We assessed model goodness-of-fit using a cutoff value of ≥ 0.95 for the comparative fit index (CFI) and the Tucker-Lewis index (TLI), and a cutoff value of ≤ 0.06 for the root mean square error of approximation (RMSEA) [16, 17]. We conducted EFA with all 11 NPEP Knowledge Scale items using the mean and variance-adjusted weighted least square (WLSMV) estimator in the first subsample to explore the underlining factor structure; the Kaiser-Guttman rule (eigenvalues > 1.0) was used for factor selection [18]. We then conducted CFA using the WLSMV estimator in the second subsample to examine the model suggested by the EFA. The CFA solution was evaluated as having acceptable overall goodness-of-fit, no focal areas of ill fit (absence of large modification indices [MI] and standardized residuals), and no out-of-range values in the parameter estimates [19]. However, there was a high correlation between two items, thus they were removed. We ran a second CFA with the remaining nine items and retained the model, given good model fit indices and reasonable interpretability.

We identified outliers in count variables (i.e., past week instances of anal sex with males and number of past six months male partners) using a robust rule ((|X-Mdn|/1.483(median absolute deviation)) > 2.24) and converted to values that equal the next highest scores that were not an outlier [19]. We then conducted differential item functioning (DIF) analyses to check any item bias using multiple indicators multiple causes modeling (MIMIC) and inspected MI and associated expected parameter change values [18]. Finally, we conducted a SEM analysis among the whole sample to estimate the relationships among NPEP knowledge, HIV knowledge, and sociodemographic and behavioral characteristics (see the supplemental file for the plot for power and sample size).

## Results

Table 1 presents findings related to socio-demographics, HIV-related risk behaviors and services utilized, and behavioral characteristics of Chinese MSM. There were no significant differences in any of these variables between participants assigned to the EFA vs. CFA subgroups (all *p*’s > 0.05).

### Factor analyses of NPEP knowledge scale

All 11 items of the NPEP Knowledge Scale were significantly correlated with each other ($\varrho$ = 0.282–0.882, *p’s* < 0.01). Results from the EFA among the subsample of 219 participants showed that only one eigenvalue was larger than one. The two-factor model did not converge. The one-factor model had acceptable fit indices (RMSEA = 0.061 [90% CI 0.038–0.082], CFI = 0.998, TLI = 0.998) and was therefore retained. Geomin rotated loadings for 11 items were all larger than 0.50 (see Table 2).

Results from the CFA with 11 items among the second subsample (with 200 participants) also indicated good fit (RMSEA = 0.033 [90% CI 0.000-0.060], CFI = 0.999, TLI = 0.999). However, Mplus warned that the sample correlations of Items C and D, and Items I and J were very high ($\varrho$= 0.992, $\varrho$ = 0.990, respectively). Given that the content of two items (C and J) were embedded in other items (i.e., Items A, D, and I), we decided to delete these two items. The removal of Items C and J also enhanced the simplicity of the scale. Results from the CFA with nine items among the second subsample also showed good fit (RMSEA = 0.040 [90% CI 0.000-0.073], CFI = 0.999, TLI = 0.998). Standardized factor loadings for each item are shown in Table 3 (item *R*^*2*^‘s = 0.527–0.969, *p*’s < 0.001). Latent variable modeling showed that the NPEP Knowledge Scale’s reliability was 0.903 (95% CI 0.891–0.915) [20].

**Table 3:** Unstandardized and standardized loading for confirmatory factor analysis model of NPEP knowledge scale among Chinese MSM, 2018 (n = 200)

Parameter estimate	Unstandardized loading (*SE*)	Standardized loading(*SE*)
NPEP → A	1.000	0.921 (0.020) ***
NPEP → B	1.068 (0.025)***	0.983 (0.013) ***
NPEP → D	1.069 (0.031) ***	0.984 (0.021) ***
NPEP → E	0.798 (0.050) ***	0.735 (0.047) ***
NPEP → F	0.941 (0.037) ***	0.866 (0.033) ***
NPEP → G	0.788 (0.053) ***	0.726 (0.050) ***
NPEP → H	0.976 (0.027) ***	0.898 (0.026) ***
NPEP → I	1.060 (0.026) ***	0.976 (0.015) ***
NPEP → K	0.896 (0.043) ***	0.825 (0.039) ***

*Note*. The CFA model has good fit indices (RMSEA = 0.040, 90% CI 0.000-0.073, CFI = 0.999, TLI = 0.998). As suggested by the modification indices, adjustment of the CFA model is not needed. Standard errors are in the parenthesis. *** p < 0.001

### DIF analysis for the NPEP knowledge scale

We next examined whether any potential factors led to DIF of the nine remaining items on the NPEP Knowledge Scale. We included all variables listed in Table 1 in the DIF analyses using MIMIC model. This model had very good fit (RMSEA = 0.017 [90% CI 0.000-0.029], CFI = 0.997, TLI = 0.997).

MI in regard to covariate-indicator direct effects suggested that the direct effects (namely, intercept DIF or item bias) of STI diagnosis on Item B (MI = 8.027), alcohol use frequency on Item A (MI = 4.966), education on Item B (MI = 4.447), and ever used illicit drugs on Item K (MI = 4.045) were above the critical value of 4.0 [18]. When these four effects were released iteratively, the model fit indices did not change, which indicates that, overall, these effects were not substantial. However, the partially standardized solution suggested that Item B showed DIF in terms of the past year STI diagnosis with a significant and large effect (β = -0.928, *p* < 0.01) [21], indicating that MSM who were diagnosed with an STI tended to choose the wrong answer for Item B regardless of their overall NPEP knowledge. In the subsequent analysis, we kept this effect relaxed to account for item bias against MSM recently diagnosed with an STI. The other three effects were small or non-significant (β = 0.122, *p* < 0.01; β = 0.174, *p* < 0.001; and β = -0.568, *p* = 0.05, respectively), indicating ignorable item bias [21].

### Structural model of NPEP knowledge, HIV knowledge, and covariates

**Fig. 1:**
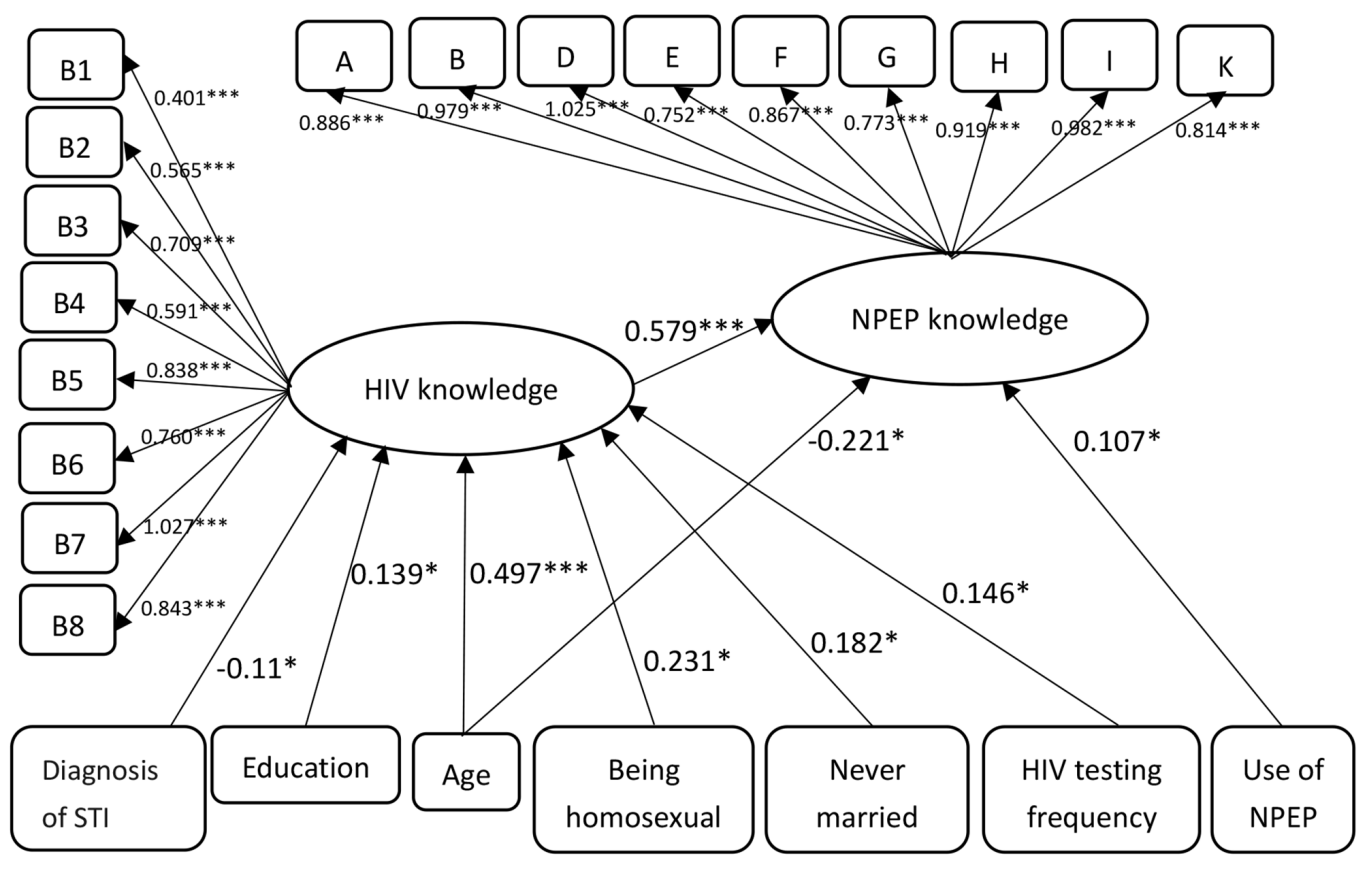
Structural equation model of HIV knowledge, NPEP Knowledge and Covariates among Chinese MSM (n = 419) *Note:* * p < 0.05, ** p < 0.01, *** p < 0.001; Fit index: RMSEA = 0.018, 90% CI 0.006–0.026, CFI = 0.993, TLI = 0.992. Only significant routes were included in the figure. All path coefficients shown were standardized

We then used SEM to examine the NPEP Knowledge Scale’s validity. As shown in Fig. 1, we modeled the NPEP knowledge score as the outcome, the HIV knowledge score as the penultimate outcome, and sociodemographic and behavioral factors (i.e., age, ethnicity, duration of local residence, education, income, marital status, sexual orientation, recent number of times having anal sex with males, number of male sexual partners, condom use during the most recent anal sex, condom rupture/slippage/intentional pull-out when having sex with other males, alcohol use frequency, STI diagnosis, HIV testing frequency, illicit drug use, and NPEP use) as predictors of both outcomes. The structural model indicated good fit to the data (RMSEA = 0.018 [90% CI 0.006–0.026], CFI = 0.993, TLI = 0.992). NPEP knowledge was positively associated with ever use of NPEP (β = 0.107, *p* < 0.05), demonstrating evidence of criterion validity. NPEP knowledge was positively associated with HIV knowledge (β = 0.579, *p* < 0.001), demonstrating evidence of convergent validity. NPEP knowledge was significantly negatively associated with age (β = -0.221, *p* < 0.05). HIV knowledge was negatively associated with STI diagnosis and positively associated with education, age, being homosexual, never being married, and HIV testing frequency (as shown in Fig. 1).

## Discussion

In this study, we systematically developed an NPEP Knowledge Scale and analyzed the scale’s factor structure, reliability, item bias, and associations with HIV knowledge and related covariates with a sample of Chinese MSM. There is a dearth of literature assessing detailed and comprehensive NPEP knowledge, particularly among MSM. Most previous studies reported NPEP awareness using a single question or item and suggest that basic NPEP awareness rates are generally quite low, not to mention a general lack of comprehensive knowledge of NPEP [22–25]. A few studies pointed out a lack of detailed NPEP knowledge as a barrier to effective NPEP implementation [26]. This literature highlights an urgent need for an empirically validated instrument that measures detailed NPEP knowledge among multiple groups, including MSM, health care providers, and individuals at high risk for HIV worldwide.

The NPEP Knowledge Scale developed in this study has excellent content, convergent, and criterion validity. In developing items, we referred to important NPEP guidelines from the United States and World Health Organization [27–29], defined and operationalized the construct of NPEP knowledge comprehensively within a Chinese context, and took into account community members’ and providers’ perceptions. The scale demonstrates good internal consistency. The unidimensional factor structure of the scale was found in EFA and confirmed in the CFA among independent subsamples, which indicates the scale was understood in the same way and had a stable structure across the two subsamples.

The DIF analyses showed that DIF does not exist or is ignorable for most of the items on the NPEP Knowledge Scale, except that Item B (“A person does not need to take NPEP after having unprotected sex with people with unknown HIV status”) was more likely to be incorrect among those diagnosed with an STI in the past 12 months, regardless of their overall scale score. This finding may be due to people who have been recently diagnosed with an STI having engaged in higher risk sexual activity, and it is understandable that their recent behavior might demonstrate that they have a weaker understanding of risk outcomes.

HIV knowledge and NPEP knowledge were significantly related, indicating good convergent validity of the NPEP Knowledge Scale. The known-groups validity (criterion validity) of the NPEP Knowledge Scale was also good: those who had ever used NPEP scored significantly higher in NPEP knowledge than those who have not used NPEP.

HIV knowledge was positively related to age, while NPEP knowledge was negatively associated with age. This discrepancy may be due to the fact that HIV knowledge has been more widely distributed through nationwide campaigns and projects, particularly among the key population of MSM; this suggestion is supported by the findings in the current study showing that most (70.4%) participants scored highly on HIV knowledge. However, NPEP is comparatively new, and about half of participants indicated no or low NPEP knowledge scores. This finding may be due to PEP not being widely accessible yet in China, and people who are potentially exposed in non-occupational settings can access PEP only on a case-by-case basis. However, younger MSM mainly use the Internet (including social media) to obtain information and connect to peers, and the Internet may have been used to facilitate younger MSM’s higher knowledge about NPEP.

The newly developed NPEP Knowledge Scale is the first of its kind, both among Chinese MSM and worldwide, and has important public health implications. This scale can be used in a wide array of settings including research and program evaluation, such as evaluating levels of NPEP knowledge before and after related interventions. This scale is brief, with nine items, and is also convenient for clinical use. Health care providers can use this scale to quickly evaluate clients’ knowledge about PEP before they introduce and/or prescribe PEP to their clients. Furthermore, CBOs serving MSM can also use this scale to evaluate patrons’ NPEP knowledge and adjust their knowledge provision for their prevention counseling services.

### Limitations

The current study is subject to limitations. First, the use of convenience sampling may limit the generalizability of the findings. Interpretations of the results should be done cautiously and take into account the sample characteristics, such as the majority of participants being younger than 35 years of age and having an education of college or above. The level of risk in this sample might be comparatively low, since we recruited a number of people who would not be candidates for NPEP with their current risk profile. In addition, this is a cross-sectional study and test-retest reliability therefore cannot be conducted, which provides no information about the temporal stability or responsiveness to change. Lastly, the external validity of this newly developed NPEP Knowledge Scale still needs to be examined among diverse groups of people, including both MSM and non-MSM populations, and across a wide array of natural and cultural environments.

## Conclusions

It is important to have access to a specific and effective tool to examine NPEP knowledge, particularly to better facilitate KAP studies with NPEP knowledge among key populations. In this study, we developed a NPEP Knowledge Scale and then tested the psychometric properties of the scale in the real world, among an often overlooked, key population of Chinese MSM. The results indicate that this newly developed scale is a valid measure of their NPEP knowledge, demonstrating very good reliability and validity. To the best of our knowledge, this is the first scale of its kind to assess detailed NPEP knowledge. This scale can contribute to NPEP-related research, program evaluation, and clinical and community services. However, additional studies are still needed in the future so as to further validate this scale. Future studies should recruit random samples among diverse populations, adopt longitudinal designs, and be conducted in multiple sites with different social and cultural profiles.

## Electronic supplementary material

Below is the link to the electronic supplementary material.

Supplementary Material 1

## Data Availability

The datasets used and/or analyzed in the current study are available from the corresponding author.
