# Supplementary material for: Development of the non-occupational post-exposure prophylaxis (NPEP) knowledge scale among Chinese men who have sex with men
Source: BMC Public Health. 2023 Jul 11;23:1329. doi: 10.1186/s12889-023-16206-5 (PMC10337112; doi:10.1186/s12889-023-16206-5)
Supplement: Supplementary file 1 — Supplementary Material 1 [file 12889_2023_16206_MOESM1_ESM.docx]

**Supplementary file: Creating a plot for power and sample size for RMSEA**

**Results from Rweb**

You are using Rweb1.03 on the server at rweb.stat.umn.edu

R version 3.0.2 (2013-09-25) -- "Frisbee Sailing"

Copyright (C) 2013 The R Foundation for Statistical Computing

Platform: x86_64-pc-linux-gnu (64-bit)

R is free software and comes with ABSOLUTELY NO WARRANTY.

You are welcome to redistribute it under certain conditions.

Type 'license()' or 'licence()' for distribution details.

R is a collaborative project with many contributors.

Type 'contributors()' for more information and

'citation()' on how to cite R or R packages in publications.

Type 'demo()' for some demos, 'help()' for on-line help, or

'help.start()' for an HTML browser interface to help.

Type 'q()' to quit R.

Rweb:> png(file= "/tmp/Rout.14597.%03d.png")

Rweb:>

Rweb:> #Power analysis for CSM

Rweb:>

Rweb:> alpha <- 0.05 #alpha level

Rweb:> d <- 388 #degrees of freedom

Rweb:> nlow <- 50 #lower sample size

Rweb:> nhigh <- 500 #upper sample size

Rweb:> step <- 10 #steps between sample size

Rweb:> rmsea0 <- 0 #null hypothesized RMSEA

Rweb:> rmseaa <- 0.05 #alternative hypothesized RMSEA

Rweb:>

Rweb:> #Code below this point need not be changed by user

Rweb:> pow1<-0

Rweb:> nseq<-seq(nlow,nhigh, by=step)

Rweb:> for(i in nseq){

+ #ii<-as.character(i)

+ ncp0 <- (i-1)*d*rmsea0^2

+ ncpa <- (i-1)*d*rmseaa^2

+ #Compute power

+ if(rmsea0<-="" qchisq(alpha,d,ncp="ncp0,lower.tail=F)" pow="" pchisq(cval,d,ncp="ncpa,lower.tail=F)" }="" if(rmsea0="">rmseaa) {

+ cval <- qchisq(1-alpha,d,ncp=ncp0,lower.tail=F)

+ pow <- 1-pchisq(cval,d,ncp=ncpa,lower.tail=F)

+ }

+ pow1<-c(pow1,pow)

+ }

Rweb:> pow1<-pow1[-1]

Rweb:> plot(nseq,pow1,xlab="Sample Size",ylab="Power",main="Compute Power for RMSEA",type="l",lwd=2,cex.axis=1.5,cex.lab=1.5,cex.main=1.5)

Rweb:>


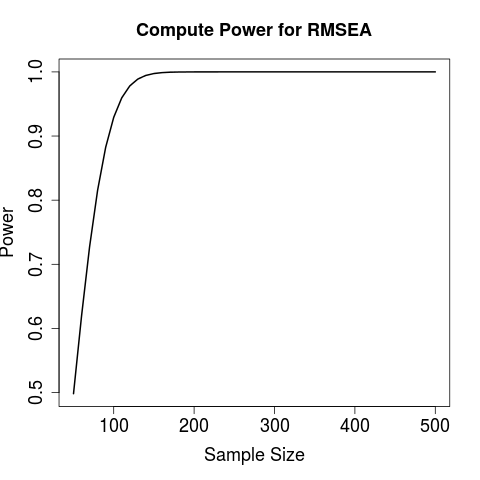


Reference:

Preacher, K.J. and Coffman, D.L., (2006). Computing power minimum sample size for RMSEA [Computer software]. Available from http://quantpsy.org/.
